# Loss of Glutamate Decarboxylase 67 in Somatostatin-Expressing Neurons Leads to Anxiety-Like Behavior and Alteration in the Akt/GSK3β Signaling Pathway

**DOI:** 10.3389/fnbeh.2019.00131

**Published:** 2019-06-18

**Authors:** Shigeo Miyata, Ryota Kumagaya, Toshikazu Kakizaki, Kazuyuki Fujihara, Kaori Wakamatsu, Yuchio Yanagawa

**Affiliations:** ^1^Department of Genetic and Behavioral Neuroscience, Graduate School of Medicine, Gunma University, Maebashi, Japan; ^2^Division of Molecular Science, Graduate School of Science and Technology, Gunma University, Kiryu, Japan

**Keywords:** GABA, GAD, somatostatin, depression, anxiety, Akt, GSK3β

## Abstract

Major depressive disorder (MDD) is a highly prevalent psychiatric disorder worldwide. Several lines of evidence suggest that the dysfunction of somatostatin (SOM) neurons is associated with the pathophysiology of MDD. Importantly, most SOM neurons are γ-aminobutyric acid (GABA) interneurons. However, whether the dysfunction of GABAergic neurotransmission from SOM neurons contributes to the pathophysiology of MDD remains elusive. To address this issue, we investigated the emotional behaviors and relevant molecular mechanism in mice lacking glutamate decarboxylase 67 (GAD67), an isoform of GABA-synthesizing enzyme, specifically in SOM neurons (SOM-GAD67 mice). The SOM-GAD67 mice exhibited anxiety-like behavior in the open-field test without an effect on locomotor activity. The SOM-GAD67 mice showed depression-like behavior in neither the forced swimming test nor the sucrose preference test. In addition, the ability to form contextual fear memory was normal in the SOM-GAD67 mice. Furthermore, the plasma corticosterone level was normal in the SOM-GAD67 mice both under baseline and stress conditions. The expression ratios of p-Akt^Ser473^/Akt and p-GSK3β^Ser9^/GSK3β were decreased in the frontal cortex of SOM-GAD67 mice. Taken together, these data suggest that the loss of GAD67 from SOM neurons may lead to the development of anxiety-like but not depression-like states mediated by modification of Akt/GSK3β activities.

## Introduction

Major depressive disorder (MDD) affects approximately 10% of the population at some point in their life and is the leading cause of physical impairment, medical comorbidity, and mortality across the world (Penninx et al., 2013; Sato and Yeh, 2013). However, the current treatments are only partially effective, and patients fail to respond to trials with existing antidepressant agents targeting the monoaminergic systems (Fekadu et al., 2009; Kupfer et al., 2012). Clarifying the molecular biology of MDD is desired for developing innovative therapeutics.

Several lines of evidence indicate that the dysfunction of somatostatin (SOM)-expressing cells is likely associated with the pathophysiology of MDD (Fee et al., 2017). In the postmortem brain of patients with MDD, the expression levels of SOM were decreased in the dorsolateral prefrontal cortex (Sibille et al., 2011), the subgenual anterior cingulate cortex (Tripp et al., 2011) and the amygdala (Guilloux et al., 2012). In animal studies, mice subjected to chronic mild stress, an animal model of depression, demonstrated a decrease in the mRNA level of SOM in the prefrontal cortex (Banasr et al., 2017). On the other hand, mice with increased excitability in SOM neurons by deletion of the γ2-subunit of γ-aminobutyric acid_A_ (GABA_A_) receptors demonstrated anti-anxiety and anti-depressive behaviors (Fuchs et al., 2017). SOM knockout (KO) mice displayed a higher response to stress in plasma corticosterone levels (Zeyda et al., 2001; Lin and Sibille, 2015; Viollet et al., 2017). SOM KO mice displayed no change in emotional behaviors (Zeyda et al., 2001; Viollet et al., 2017) or mild anxiety-like behavior (Lin and Sibille, 2015). Lin and Sibille reported the anxiety-like/depression-like behaviors were pronounced after exposure to chronic mild stress (Lin and Sibille, 2015).

Importantly, most SOM-expressing cells in the central nervous system are GABA interneurons (Kosaka et al., 1988; Kubota et al., 1994; Esclapez and Houser, 1995; Gonchar and Burkhalter, 1997; Uematsu et al., 2008). Neuroimaging studies have demonstrated a reduction in GABA levels in the brains of patients with MDD (Sanacora et al., 1999; Hasler et al., 2007). GABA is synthesized from glutamate by glutamate decarboxylase (GAD). GAD exists in two isoforms, GAD67 and GAD65, which are independently encoded by the *GAD1* and *GAD2* genes, respectively (Soghomonian and Martin, 1998; Ji et al., 1999). Several studies have demonstrated decreased expressions of GAD67 but not GAD65 in the postmortem brains of patients with MDD (Karolewicz et al., 2010; Scifo et al., 2018), although these changes were not observed by others (Pehrson and Sanchez, 2015). Therefore, the emotional disabilities in patients with MDD may be associated with the dysfunction of GABAergic neurotransmission from SOM neurons, which disrupts an inhibitory control to neural excitability (Fee et al., 2017). Global GAD67 KO mice show cleft palate and omphalocele, and all of them die during the first day after birth (Asada et al., 1997; Kakizaki et al., 2015). We recently developed mice with conditional KO of GAD67 specifically in parvalbumin (PV)-expressing cells (PV-GAD67 mice) or SOM-expressing cells (SOM-GAD67 mice). The PV-GAD67 mice demonstrated oscillational disturbance across cortical layers and schizophrenia-like behavioral abnormalities (Fujihara et al., 2015; Kuki et al., 2015). However, we had yet to investigate the behavioral phenotypes of the SOM-GAD67 mice. Behavioral examination of SOM-GAD67 mice is important for clarifying whether the deficiency of GAD67-mediated GABA in SOM neurons contributes to MDD-related symptoms.

Akt and glycogen synthase kinase-3 β-isoform (GSK3β) are serine/threonine protein kinases that regulate multiple cellular functions including neuroplasticity and cell survival (Descorbeth et al., 2018; Wu et al., 2018). Akt/GSK3β signaling is an important signal that regulates emotional behaviors in rodents (Sui et al., 2008; Bali and Jaggi, 2016; Pan et al., 2016; Slouzkey and Maroun, 2016). Recently, the Akt/GSK3β pathway has attracted attention in the molecular biology of MDD and as a novel target of therapeutic agents (Kitagishi et al., 2012). Interestingly, GABA signaling affects Akt/GSK3β activities (Lu et al., 2012). Therefore, the functional alteration of SOM-expressing GABA neurons may affect Akt/GSK3β activities in the brain.

The aim of this study was to resolve the role of GAD67 in SOM neurons on emotional regulation using SOM-GAD67 mice. We also examined the plasma corticosterone levels and the expression levels of Akt and GSK3β proteins, which are relevant molecules to the pathophysiology of MDD.

## Materials and Methods

### Ethics Statement

This study was performed in accordance with the Guidelines for Animal Experimentation at Gunma University Graduate School of Medicine and was approved by the Gunma University Ethics Committee (Permit number: 14-006). Every effort was made to minimize the number of animals used and their suffering.

### Animals

We previously reported the generation of SOM-GAD67 mice (Kuki et al., 2015). Briefly, SOM-IRES-Cre mice (Taniguchi et al., 2011) were obtained from Jackson Laboratories (Bar Harbor, ME, USA; Stock No: 028864), and GAD67-floxed mice were previously described (Obata et al., 2008; Fujihara et al., 2015). SOM^IRES−Cre/+^;GAD67^flox/flox^ mice (SOM-GAD67 mice) were obtained by crossing female GAD67^flox/flox^ mice and male SOM^IRES−Cre/+^;GAD67^flox/+^ mice. The littermate GAD67^flox/flox^ mice were used as the control. We only used male mice from 8 weeks to 16 weeks of age for the behavioral tests, enzyme immunoassay and western blottings. The animals were housed with 2–3 mice per cage [16.5 × 27 × 12.5 (H) cm] and had free access to food and water. The animal rooms for breeding and experiments were maintained at 22 ± 3°C with a 12-h light-dark cycle (lights on at 6:00, lights off at 18:00). The animals were used only once.

### Genotyping

Genotyping of the transgenic mice was performed by PCR using tail genomic DNA. The primer sequences were as follows: Cre allele, 5′-GTCTCTGGTGTAGCTGATGATCCGAA-3′ and 5′-CCCTGTTTCACTATCCAGGTTACGGA-3′; GAD67 allele, 5′-ACCTTGGCAGCTAACTAGGAGGA-3′ and 5′-ACAGATCGGATGGGGAAGCATAA-3′. The lengths of the amplified DNA fragments were as follows: Cre allele, 321 bp; GAD67 allele, 155 bp; loxP-inserted GAD67 allele, 258 bp.

### Open-Field Test

Each mouse was placed in the center of an open-field apparatus [50 cm × 50 cm × 40 (H) cm] that was illuminated by light-emitting diodes (30 lux at the center of the field) and allowed to move freely for 5 min. The time spent in the central area of the field (36% of the field) was recorded as the index of interest. The data were collected and analyzed using ImageJ OF4 (O’Hara & Co., Ltd., Tokyo, Japan), which is a modified software that is also based on the public domain ImageJ program. The procedure was referenced to our previous report (Miyata et al., 2016).

### Contextual Conditioned Fear Test

Training and testing took place in a chamber [10 × 10 × 12 (H) cm] equipped with a grid floor placed in an acoustic box. The grid floor was wired to an isolated shock generator (MSG-001, Toyo Sangyo Co. Ltd., Japan). The experiments were conducted over the course of two consecutive days. On day 1 (training session), mice were individually placed in the chamber and received scrambled foot-shocks (either 0.2 mA or 0.4 mA, 2 s) at pseudo-random times; 2.5, 5, 9 and 11.5 min, after the start of the session. Thirty seconds after the last foot shock, the mice were returned to the home cage. Twenty-four hours later, the mice were placed in the same chamber as on day 1 for 6 min without foot-shock exposure (test session). Background white-noise (55 dB) was presented during both the training and the test sessions. The protocol was based on the first two consecutive days of Lattal’s method (Lattal et al., 2007) to test contextual fear conditioning. Mouse behavior was recorded and analyzed using Time FZ1 for Contextual and Cued Fear Conditioning Test software (O’Hara & Co., Ltd.). The percentage of duration of freezing behavior in the test session was calculated and compared between the genotypes.

### Forced Swimming Test

Each mouse was placed in an acrylic cylinder (22 cm in height, 11.5 cm in diameter) containing 15 cm of water at room temperature (22 ± 3°C). The cylinder was placed in an isolation box. The behavior of each mouse was recorded for 6 min using a CCD camera connected to a personal computer and analyzed using ImageJ PS1 (O’Hara & Co., Ltd.), which is a modified software package that is based on the public domain ImageJ program (developed at the U.S. National Institutes of Health and available at: http://rsb.info.nih.gov/ij). The procedure was the same as that referenced in our previous report (Miyata et al., 2016). The percentage of time spent immobile during the 6-min period was calculated and compared between the genotypes.

### Sucrose Preference Test

Sucrose preference test is a well-accepted behavioral test measuring an anhedonia-like state of mice and rats (Katz, 1982; Willner, 1997). Mice preferentially take sweet-taste solution compared with water. The sweet-taste preference disappears in model mice of depression, such as mice subjected to chronic mild stress. This behavioral phenotype disappears by sub-chronic treatment with antidepressant agents (Willner et al., 1987).

One week before the measurement, the mice were provided 2% sucrose solution in a drinking bottle for 24 h to habituate to sweet taste. Each mouse was subjected to water deprivation for 16 h before starting the measurement. Mice were transferred to an individual cage [16.5 × 27 × 12.5 (H) cm], and then two preweighted bottles (one containing tap water and another containing 2% sucrose solution) were presented to each mouse for 4 h. The bottles were weighed again, and the weight difference was considered to be the mouse intake from each bottle. The sum of water and sucrose intake was defined as the total intake, and sucrose preference was expressed as the percentage of sucrose intake from total intake.

### Plasma Corticosterone

Blood samples were obtained from the tail vein by a small incision. Immediately after the initial sampling, the mice were restrained in 50-mL Falcon® tubes with air vents for 120 min. The blood samples were collected at 15 and 120 min during the restraint stress. After the cessation of restraint stress, the mice were returned to their home cage. Sixty minutes later, the final blood sampling was conducted. The blood samples were centrifuged at 1,000 *g* for 10 min at 4°C, and the plasma samples were collected and stored at −80°C until analysis. Blood sampling was conducted between 9:00 and 13:00 on the day of the experiment.

Plasma corticosterone concentrations were determined using a commercially available enzyme immunoassay kit (Enzo Life Sciences, Inc., Farmingdale, NY, USA) following the manufacturer’s instructions.

### Western Blot

The mice were killed by decapitation. The frontal cortex (FCx) was quickly dissected on an ice-cold stainless plate, immediately frozen in liquid nitrogen and stored at −80°C until use. The dissection was performed according to the Chiu’s study (Chiu et al., 2007). The tissues were homogenized in ice-cold buffered sucrose (0.32 M) solution containing 20 mM Tris-HCl (pH 7.5), protease inhibitor cocktail (P8340, Sigma-Aldrich, Inc.) and phosphatase inhibitor cocktail (07575-51, Nacalai Tesque, Inc.). The homogenates were centrifuged at 1,000 *g* for 10 min at 4°C, and the supernatants were collected as the protein samples (S1 fraction). The protein concentrations were determined using a TaKaRa BCA Protein Assay Kit (T9300A, Takara Bio Inc., Japan).

The protein samples were diluted with electrophoresis sample buffer. Proteins (15 μg) were separated by SDS-polyacrylamide gels and transferred to a PVDF membrane. Blots were probed with antibodies to Akt (pan; 1:1,000, #4691, Cell Signaling Technology Japan, K.K.), phospho-Akt (Ser473; 1:2,000, #4060, Cell Signaling Technology, Danvers, MA, USA), phospho-Akt (Thr308; 1:1,000, #13038, Cell Signaling Technology, Danvers, MA, USA), GSK3β (1:1,000, #9832, Cell Signaling Technology, Danvers, MA, USA), phospho-GSK3β (Ser9; 1:1,000, #5558, Cell Signaling Technology, Danvers, MA, USA) and β-actin (1:4,000, M177-3, Medical and Biological Laboratories Co. Ltd., Japan). Immunoblots were developed using horseradish peroxidase-conjugated secondary antibodies (GE Healthcare) and then detected with chemiluminescence reagents (ECL prime, GE Healthcare) and visualized by an Light Capture AE-9672 (ATTO Co., Ltd.). The density of the bands was determined using ImageJ software.

The Akt and GSK3β activities were assessed by calculating the ratio of the band densities of phosphorylated/total proteins. The band densities of β-actin were used as the loading control.

### Data Analysis

Statistical analyses were conducted using BellCurve for Excel ver. 2.12 (Social Survey Research Information Co., Ltd., Tokyo, Japan). Significant differences between two groups were evaluated by Student’s *t*-test. Significant differences among the multiple groups were analyzed by one-way and two-way analysis of variance (ANOVA) with a Bonferroni multiple comparison test. Statistical significance was defined as a *p*-value less than 0.05. The data were expressed as means ± SE.

## Results

### Behavioral Phenotypes of SOM-GAD67 Mice

**Figure 1:**
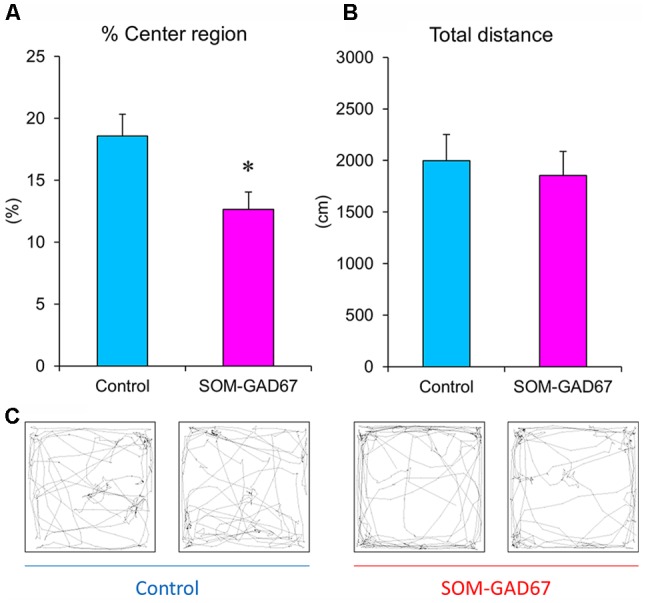
Exploratory behaviors of the somatostatin (SOM)-glutamate decarboxylase 67 (GAD67) mice (*n* = 8) and control mice (*n* = 8) in the open-field test. The percent exploration of the center field **(A)** and total path length **(B)** for 5 min. **(C)** Illustrative examples of the travel pathway in two control and two SOM-GAD67 mice. Data represent the means + SE. *p < 0.05 vs. the control mice (Student’s *t*-test).

To assess the anxiety-like state of mice, we performed the open-field test. The SOM-GAD67 mice exhibited significantly less time spent in the center field than the control mice (Figures 1A,C), but the total path length in the open-field test did not differ between the genotypes (Figures 1B,C).

**Figure 2:**
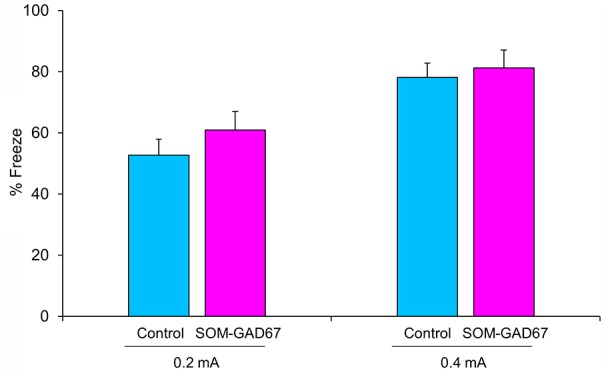
Freezing behavior of the SOM-GAD67 mice and control mice in the contextual conditioned fear test. The mice were subjected to either 0.2 mA (SOM-GAD67; *n* = 7, control; *n* = 7) or 0.4 mA (SOM-GAD67; *n* = 6, control; *n* = 4) foot-shocks in the conditioning session. Twenty-four hours later, the mice were returned to the same chamber, and freezing behavior was measured for 6 min without foot-shock presentation. The percent duration of freezing is shown. Data represent the means + SE.

Next, we evaluated the ability to form fear memory in mice in the contextual conditioned fear test. The duration of freezing behavior observed in the test session was not significantly different between the genotypes (Figure 2).

**Figure 3:**
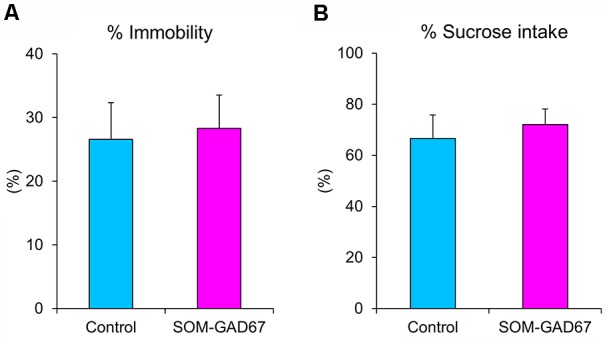
Depression-like behaviors of the SOM-GAD67 mice and control mice in the forced swimming test and the sucrose preference test. **(A)** The percentage of time spent immobile in the forced swimming test is shown (SOM-GAD67; *n* = 9, control; *n* = 9). **(B)** The percent intake of sucrose in the sucrose preference test is shown (SOM-GAD67; *n* = 8, control; *n* = 7). Data represent the means + SE.

We further evaluated the depression-like state in mice in the forced swimming test and the sucrose preference test. In the forced swimming test, the duration of immobility was not significantly different between the genotypes (Figure 3A). In addition, there was also no difference in sucrose preference between the genotypes (Figure 3B).

### Stress Reactivity in SOM-GAD67 Mice

**Figure 4:**
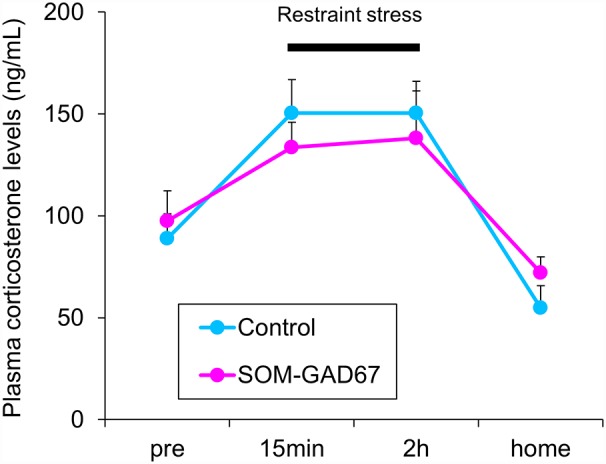
Plasma corticosterone levels in the SOM-GAD67 mice (*n* = 7) and control mice (*n* = 7). After the first blood sample collection (pre), the mice were subjected to restraint stress for 120 min. The blood was collected 15 min and 120 min after the beginning of the stress exposure. After the cessation of the stress exposure, the mice were returned to their home cage, and 60 min later, the final blood sample was collected. The plasma corticosterone levels were determined by the enzyme immunoassay kit. Data represent the means + SE.

To assess the reactivity to stress, we measured the plasma corticosterone levels in mice under baseline conditions and during a stressful condition. Under baseline, there was no difference in the plasma corticosterone levels between the genotypes. Under conditions of restraint stress, the plasma corticosterone levels were increased in both genotypes, but the elevated plasma corticosterone levels were similar between the genotypes. One hour after cessation of the stress, the plasma corticosterone levels had decreased in both genotypes, but there were no differences in the plasma corticosterone levels between the genotypes (Figure 4).

### Cortical Akt/GSK3β Signaling in SOM-GAD67 Mice

**Figure 5:**
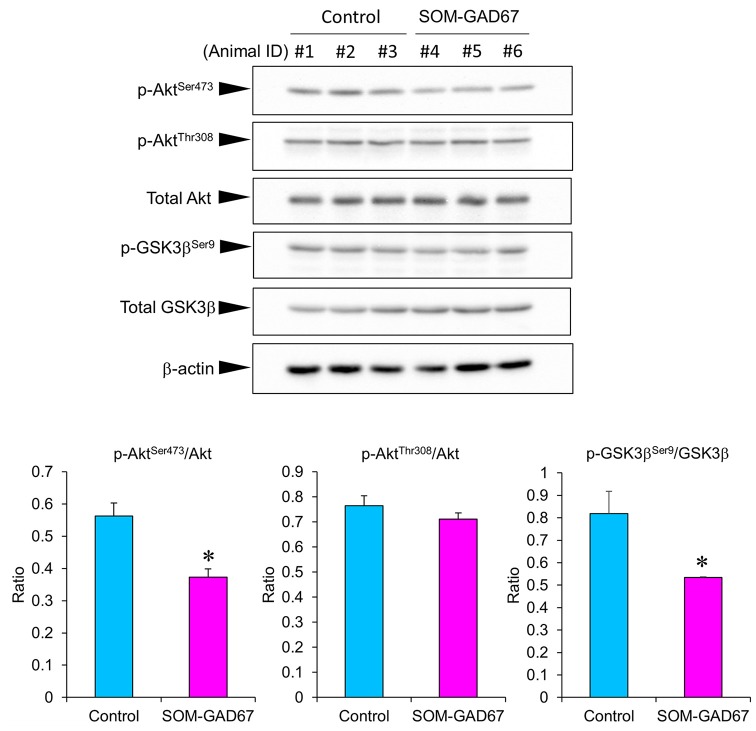
Protein levels of Akt and glycogen synthase kinase-3 β (GSK3β) in the frontal cortex of the SOM-GAD67 mice (*n* = 3) and control mice (*n* = 3). The band densities were determined using ImageJ software. The expression ratio of phosphorylated/total proteins was compared between the genotypes. Data represent the means + SE. *p < 0.05 vs. the control mice (Student’s *t*-test).

To assess the link between Akt/GSK3β signaling and anxiety-like behavior in SOM-GAD67 mice, we examined the expression levels of Akt and GSK3β by Western blotting and calculated the expression ratio of phosphorylated forms/total proteins. The expression ratio of the p-Akt^Ser473^/Akt protein was significantly lower in the FCx of the SOM-GAD67 mice than in that of the control mice (Figure 5). On the other hand, the expression ratio of the p-Akt^Thr308^/Akt protein was not different between the genotypes. The expression ratio of p-GSK3β^Ser9^/GSK-3β protein was significantly lower in the SOM-GAD67 mice than in the control mice (Figure 5).

## Discussion

In the current study, SOM-GAD67 mice exhibited behavioral abnormalities in the open-field test but no effects on locomotor activity. The reduction in exploration of the center region of the open field is accepted as anxiety-like behavior in rodents (Parks et al., 1998). Therefore, the deletion of GAD67 from SOM-expressing GABA neurons induced an anxiogenic-like effect in mice. In the contextual conditioned fear test, the duration of freezing was not different between the genotypes, indicating that the ability to form contextual fear memory is normal in SOM-GAD67 mice. Unexpectedly, the SOM-GAD67 mice showed depression-like behavior in neither the forced swimming test nor the sucrose preference test. The forced swimming test and the sucrose (or saccharine) preference test are accepted as animal models of depression and screening methods for antidepressant agents (Porsolt et al., 1977; Katz, 1982). Therefore, the deletion of GAD67 from SOM-expressing GABA neurons had no effect on the depression-like state in mice. Based on the current behavioral studies, we suggest that GAD67 in SOM neurons mainly regulates the anxiety-like state in mice.

In clinical studies, elevated concentrations of cortisol, the end product of the hypothalamic-pituitary-adrenal axis, are observed in the blood of patients with MDD (Schlesser et al., 1980). In addition, the blood concentrations of corticosterone, the major glucocorticoid in rodents, are increased in animal models of depression (Marcilhac et al., 1999; Miyata et al., 2004; Kubera et al., 2013; Iñiguez et al., 2014). We measured the plasma levels of corticosterone in the SOM-GAD67 mice before, during and after the restraint stress. Both genotypes demonstrated a stress-elicited increase in plasma corticosterone levels, but there was no difference between the genotypes at any time period measured. The results indicate that deletion of GAD67 from SOM neurons is not sufficient to affect the reactivity of the hypothalamic-pituitary-adrenal axis. SOM KO mice have been reported to exhibit an enhanced response to stress in plasma corticosterone levels (Zeyda et al., 2001; Lin and Sibille, 2015; Viollet et al., 2017). The hormonal reaction in the current SOM-GAD67 mice was different from that in SOM KO mice. SOM is coreleased with GABA to inhibit excitatory synaptic transmission (Martel et al., 2012). These findings indicate that the effects of SOM and GABA released from SOM neurons on the reactivity of the hypothalamic-pituitary-adrenal axis may be different.

Akt and GSK3β are serine/threonine protein kinases that regulate multiple cellular functions, including neuroplasticity and cell survival (Descorbeth et al., 2018; Wu et al., 2018). The phosphorylation of Akt at the Thr308 and Ser473 sites is needed for its full activation (Bellacosa et al., 1998). Akt at Thr308 is phosphorylated by phosphoinositide-dependent protein kinase-1 (Song et al., 2005). Akt at Ser473 is autophosphorylated or phosphorylated by mechanistic target of rapamycin complex-2 (Toker and Newton, 2000; Jacinto et al., 2006). Akt phosphorylates GSK3β at Ser9 and inhibits its kinase activity (Sutherland et al., 1993; Cross et al., 1995). Decreased activity of Akt and increased activity of GSK3β have been found in the prefrontal and occipital cortex of suicide victims with depressive disorder (Hsiung et al., 2003; Karege et al., 2007, 2011). In addition, Akt/GSK3β signaling is associated with the treatment responses of therapeutic agents of mental illness (Beaulieu et al., 2009; Kim et al., 2009; Zhang et al., 2010; Kitagishi et al., 2012; Costemale-Lacoste et al., 2016). In animal studies, mice lacking Akt2, an isoform of Akt, exhibited anxiety-like and depression-like behaviors (Leibrock et al., 2013). The expression levels of p-Akt^Ser473^ and the p-Akt^Ser473^/Akt ratio were decreased in the hippocampus in animal models of depression (Xia et al., 2016; Wu et al., 2017). In addition, the expression level of p-GSK3β^Ser9^ in the frontal cortex was decreased in an animal model of depression (Szymańska et al., 2009). Treatments with antidepressant agents normalized these alterations in p-Akt^Ser473^ and p-GSK3^βSer9^ expression in the prefrontal cortex and the hippocampus of those models (Xia et al., 2016; Szymańska et al., 2009; Wu et al., 2017). Furthermore, treatment with a GSK3β inhibitor ameliorated stress-elicited anxiety-like behavior in mice (Bali and Jaggi, 2017). In contrast, treatment with an Akt inhibitor interfered with the neuroprotective and anxiolytic-like effects of therapeutic agents (Pei et al., 2016). These findings indicate that the decreased activity of Akt and the increased activity of GSK3β contribute to the development of anxiety-like and depression-like states in rodents. In the current study, SOM-GAD67 mice demonstrated reductions in the p-Akt^Ser473^/Akt ratio and p-GSK3β^Ser9^/GSK3β ratios in the FCx, indicating that Akt kinase activity is decreased and GSK3β kinase activity is increased in the FCx of SOM-GAD67 mice, which are similar to the findings in the postmortem brain of depressive patients. The details of the mechanisms by which GAD67 in SOM neurons regulates Akt/GSK3β activity are still unclear, but impairment of Akt/GSK3β signaling may be associated with the development of an anxiety-like state in SOM-GAD67 mice. Further studies are needed to resolve this relationship.

In this study, SOM-GAD67 mice demonstrated anxiety-like behavior but not depression-like behavior. In psychiatric examinations, subsyndromal anxiety is often comorbid in patients with MDD (Zimmerman et al., 2000; Zbozinek et al., 2012; Steenkamp et al., 2017). Therefore, dysfunction of GAD67 in SOM neurons might be associated with subsyndromal anxiety but not depressive symptoms in patients with MDD.

## Conclusion

GAD67 in SOM neurons regulates the anxiety-like state in mice mediated by the modification of cortical Akt/GSK3β activity.

## Data Availability

All datasets generated for this study are included in the manuscript.

## Ethics Statement

This study was performed in accordance with the Guidelines for Animal Experimentation at Gunma University Graduate School of Medicine and was approved by the Gunma University Ethics Committee (Permit number: 14-006).

## Author Contributions

YY supervised the research. SM designed the experimental protocol, conducted the data analysis, interpretation and wrote the first draft of the manuscript. SM, RK and TK carried out animal studies including animal care. KF and KW provided critical suggestions for the research. All authors contributed to and approved the final manuscript.

## Conflict of Interest Statement

The authors declare that the research was conducted in the absence of any commercial or financial relationships that could be construed as a potential conflict of interest.
